# Application of Artificial Intelligence in Food Industry—a Guideline

**DOI:** 10.1007/s12393-021-09290-z

**Published:** 2021-08-09

**Authors:** Nidhi Rajesh Mavani, Jarinah Mohd Ali, Suhaili Othman, M. A. Hussain, Haslaniza Hashim, Norliza Abd Rahman

**Affiliations:** 1 Department of Chemical and Process Engineering, Faculty of Engineering & Built Environment, Universiti Kebangsaan Malaysia, UKM, Selangor 43600 Bangi, Malaysia; 2 Department of Biological and Agricultural Engineering, Faculty of Engineering, Universiti Putra Malaysia, UPM Serdang, 43400 Selangor, Malaysia; 3 Department of Chemical Engineering, Faculty of Engineering, University of Malaya, 50603 Kuala Lumpur, Malaysia; 4 Department of Food Sciences, Faculty of Science & Technology, Universiti Kebangsaan Malaysia, UKM, Selangor 43600 Bangi, Malaysia

**Keywords:** AI in food industry, Review, NIR, Food sensors, Model development guidelines

## Abstract

Artificial intelligence (AI) has embodied the recent technology in the food industry over the past few decades due to the rising of food demands in line with the increasing of the world population. The capability of the said intelligent systems in various tasks such as food quality determination, control tools, classification of food, and prediction purposes has intensified their demand in the food industry. Therefore, this paper reviews those diverse applications in comparing their advantages, limitations, and formulations as a guideline for selecting the most appropriate methods in enhancing future AI- and food industry–related developments. Furthermore, the integration of this system with other devices such as electronic nose, electronic tongue, computer vision system, and near infrared spectroscopy (NIR) is also emphasized, all of which will benefit both the industry players and consumers**.**

## Introduction

Artificial intelligence (AI) is defined as a field in computer science that imitates human thinking processes, learning ability, and storage of knowledge [1, 2]. AI can be categorized into two types which are strong AI and weak AI. The weak AI principle is to construct the machine to act as an intelligent unit where it mimics the human judgments, while the strong AI principle states that the machine can actually represent the human mind [3]. However, strong AI does not exist yet and the study on this AI is still in progress. The gaming industry, weather forecasting, heavy industry, process industry, food industry, medical industry, data mining, stem cells, and knowledge representation are among the areas that have been utilizing AI methods [4–11]. AI has a variety of algorithms to choose from such as reinforcement learning, expert system, fuzzy logic (FL), swarm intelligence, Turing test, cognitive science, artificial neural network (ANN), and logic programming [3]. The alluring performance of AI has made it the most favorable tool to apply in industries including decision making and process estimation aiming at overall cost reduction, quality enhancement, and profitability improvement [7, 12].

As the population in the world is rising, food demand is predicted to rise from 59 to 98% by 2050 [13]. Thus, to cater for this food demand, AI has been applied such as in management of the supply chain, food sorting, production development, food quality improvement, and proper industrial hygiene [14–16]. Sharma stated that the food processing and handling industries are expected to grow about CAGR of 5% at least until 2021 [15]. ANN has been used as a tool in aiding real complex problem solving in the food industry according to Funes and coworkers [17], while based on Correa et al., the classification and prediction of parameters are simpler when using ANN, which leads to higher usage demand of ANN over the past years [18]. Besides, FL and ANN have also acted as controllers in ensuring food safety, quality control, yield increment, and production cost reduction [19, 20]. AI technologies have also known to be beneficial in food drying technology and as process control for the drying process [21–23].

Previous studies have shown many usages of AI in food industries focusing on individual target and aims. A study has been conducted on the various ANN applications in food process modeling where it has only highlighted the food process modeling using ANN [24]. Apart from that, the implementation of AI such as ANN, FL, and expert system in food industries have been reviewed but specifically focusing on the drying of fresh fruits [23]. A review has been conducted on how food safety has been one of the main concerns in the food industry which leads to the development of smart packaging systems to fulfill the requirements of the food supply chain. Intelligent packaging monitors the condition of foods to give details on the quality of the food during storage and transportation [25]. Another study reviewed on intelligent packaging as a tool to minimize food waste where about 45 recent advances in the field of optical systems for freshness monitoring have been reported. Meat, fish products, fruits, and vegetables were covered in the study as they are the most representative fields of application [25]. Few different studies have been conducted on intelligent packaging, and these studies proved that the usage of intelligent packaging systems plays an important role in the food factory in the context of the food chain as they are able to monitor the freshness of food products and crops [23, 26–30].

There are also several other studies that have been conducted on the application of AI and sensors in food; however, the coverage is rather limited. Therefore, a comprehensive review that assembles all AI applications in the food industry as well as its combinations with appropriate sensor will be a great advantage, all of which are unavailable as to the knowledge of the author. Such review will assist in gathering the advantages, limitations, and methodologies as a one-stop guideline and reference for food industry players, practitioners, and academicians. To be exact, different types of AI and their recent application in food industries will be highlighted which comprises several AI techniques including expert system, fuzzy logic, ANN, and machine learning. In addition, the integration of AI with electronic nose (E-nose), electronic tongue (E-tongue), near infrared spectroscopy (NIRS), and computer vision system (CVS) is also provided. This paper is organized as follows. The introduction of AI is explained in the first section followed by the application of different types of AI in the food industry. Following that, the fusion of the AI with the external sensors in the food industry is presented. In the latter part, a critical review is conducted where discussion on the main application of the AI algorithms in the food industry is carried out. A flowchart is presented to assist the researchers on establishing the most appropriate AI model based on their specific case study. Then, the trends on the application of AI in the food industry are illustrated after that section. Finally, a brief conclusion is discussed in this paper.

### AI in Food Industry

The application of AI in the food industry has been growing for years due to various reasons such as food sorting, classification and prediction of the parameters, quality control, and food safety. Expert system, fuzzy logic, ANN, adaptive neuro-fuzzy inference system (ANFIS), and machine learning are among the popular techniques that have been utilized in the food industries. Prior to AI implementation, studies related to food have been going on over the years to educate the public about food knowledge as well as to improve the final outcomes related to food properties and the production of foods [31–36]. A lot of benefits can be obtained by using the AI method, and its implementation in the food industry has been going on since decades ago and has been increasing till today [37–39, 31, 32]. Nevertheless, this paper will focus on the application of AI in food industries from the year 2015 onwards since tremendous increase and innovation are seen in the implementation recently. It is worth noting that several methods such as partial least square, gastrointestinal unified theoretical framework, in silico models, empirical models, sparse regression, successive projections algorithms, and competitive adaptive reweighted sampling which have been used for prediction and enhancement of the food industries are not discussed here; instead it is narrowed down to the wide application of AI in the food industry.

#### Knowledge-based Expert System in Food Industry

**Fig. 1:**
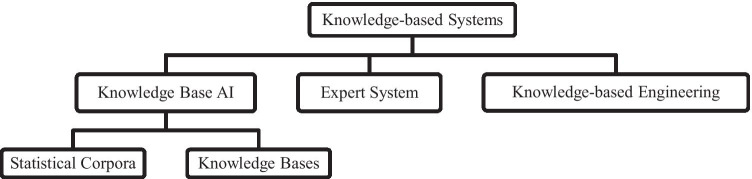
Knowledge-based system

**Fig. 2:**
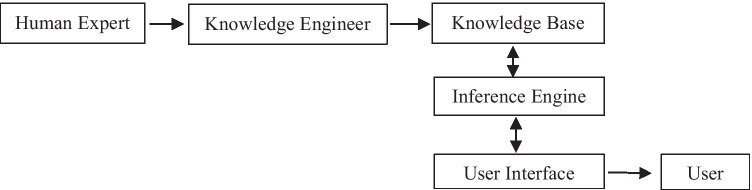
Expert system

The knowledge-based system is a computer program that utilizes knowledge from different sources, information, and data to solve complicated problems. It can be classified into three categories which are expert systems, knowledge-based artificial intelligence, and knowledge-based engineering. The breakdown of the knowledge-based system is presented in Fig. 1. The knowledge-based expert system which is widely used in the industries is a decisive and collective computer system that is able to imitate the decision-making ability of human expert [40]. It is a type of knowledge-based system that is known as among the first successful AI models. This system depends on experts for solving the complicated issues in a particular domain. It has two sub-systems, which are knowledge base and inference engine. The facts about the world are stored in the knowledge base, and the inference engine represents the rules and conditions regarding the world which are usually expressed in terms of the IF–THEN rules [41]. Normally, it is able to resolve complicated issues by the aid of a human expert. This system is based on the knowledge from the experts. The main components of the expert system (ES) are human expert, knowledge engineer, knowledge base, inference engine, user interface, and the user. The flow of the expert system is shown in Fig. 2.

The food industry has been utilizing ES for various objectives as this system is proven to be useful especially in the decision-making process. The knowledge-based expert system has been applied in white winemaking during the fermentation process for the supervision, intelligent control, and data recovery [42]. Apart from that, a web-based application was developed by implementing the ES to calculate the nutritional value of the food for the users, and the development of ES was able to help the SMIs in obtaining the details required for the qualification in obtaining the food production certificates [43]. Food safety is very important in the food industry,thus, the application of ES that is linked closely to food safety has been used extensively ranging from process design, safety management, quality of food, and risk assessment [44]. Furthermore, a prototype information technology tool and guidelines with corrective actions that considered ES in the model were developed for the food industry where few essential factors such as food safety, nutrition, quality, and cost were studied [45]. In addition, a digital learning tool, namely, MESTRAL, was developed to assist people in food processing by using models developed from research in food science and technology and simulators. This tool is based on the knowledge engineering and reflected real applications which can be mapped with the system scale and knowledge frameworks [46]. A comprehensive review was conducted by Leo Kumar on the application of the knowledge-based expert system in manufacturing planning. The paper has also discussed the utilization of ES in decision making in three wide areas which are the process planning activities, diverse applications, and manufacturing planning [41]. Moreover, Table 1 gathers some of the recent application of ES in the food industry ranging from the raw material to the final production as well as the food safety.

**Table 1:** Application of expert system in food industry

Application	Objective	Category in the industry	Important outcomes	References
Banana	To identify the banana disease and methods to overcome them	Raw material/agriculture	(i) The system was able to diagnose the diseases in the plants based on the stems, leaves, and roots which assists in preventing from the diseases happening	Budiyanto et al. [133]
Barley	To classify the barley grains	Quality control	(i) The classification accuracy by using the ES method was above 72% which was considered satisfactory	Szturo & Szczypinski [40]
Coffee	To determine the appropriate process on the dry mill	Production/quality control	(i) ES was able to select the correct production process for a specific coffee with an accuracy of 93%	Hernández-Vera et al. [134]
Coffee beans	To identify the quality grading of the coffee beans	Sensory evaluation	(i) The developed fuzzy ES, namely, AI Cupper, was able to grade the coffee with an accuracy of 95%	Livio & Hodhod [135]
Corn	To detect the corn pests and diseases	Agriculture/raw material	(i) The system was able to detect the pests and disease with an accuracy of 76.6% and also provide ways to control them (ii) The developed system was able to provide facility explanation regarding the diagnosis results	Sumaryanti et al. [136]
Food additives	To identify the halal safety rating for food additives	Food dafety	(i) The developed Halal Food Additive using ES provides the consumers with safety rating key according to their past food consumption record experience (ii) It provides consumers in finding nutritional foods and at the same time fulfilling the Halalan-Toyyiban criteria	Zakaria et al. [137]
Food products	To monitor and forecast the product quality in the production process	Production/food quality	(i) An intelligent ES was developed which is able to monitor the product quality indicators and make changes to the existing methods; recommendations for the products and the defects for the final product can be identified by this system	Blagoveshchenskiy et al. [138]
Fresh food	To optimize the distribution networks of the fresh foods	Sustainability	(i) The proposed expert system, Food Distribution Planner, was able to develop the most effective distribution method which reduces the emission of carbon dioxide by 9.6%, increase of 2.7% in operating cost, and no waste produced during the delivery time due to the preservation method utilized during the shipment	Bortolini et al. [139]
Livestock production (milk, meat)	To analyze the outcome of various variables on the performance of the livestock production	Production/raw material	(i) ES can be utilized as a decision support system for livestock producers for identifying the best practices for the livestock which maximizes the production of the meat and milk (ii) The greatest impact on the production is the type of grazing being fed to the cattle as the diet affects the health of the cow who produces the milk and the meat	Vásquez et al. [140]
Red wine and rum	To forecast the key aroma compounds for foods without using the human olfactory system	Sensory evaluation/quality control	(i) The developed rapid method system, Sensomics Based Expert System, resulted in a good agreement in the key odorants for the food aroma distillate	Nicolotti et al. [141]
Rice crops	To aid the farmers in making decisions for the rice crops	Agriculture/ production	(i) The farmers are assisted by the ES in selecting the seeds and tackling the pests and diseases for the rice crops which eventually will improve the production of the rice crops	Kharisma et al. [142]
Soybean	To identify the diseases on soybeans	Agriculture	(i) The study successfully designed an ES to identify soybean diseases by comparing the accuracy using the frame-based representation and rule-based representation method (ii) Frame-based representation ES has shown a higher accuracy compared to the rule-based ES	Rajendra et al. [143]
White winemaking	To develop a knowledge-based ES for the alcoholic fermentation process of the white winemaking	Processing/sustainability	(i) A cost-efficiency advanced control system through the knowledge-based ES was developed for the alcoholic fermentation process which was used for the supervision, control, and data recovery software of the bioreactor (ii) It was proven to be applied in winemaking at the industrial scale and can be adjusted for few areas in the food manufacturing sectors	Sipos [42]
Wine	To measure the environmental impact of viticulture at wine estate	Sustainability	(i) ES integrated with the geographic information system software was able to measure the environmental impact of viticulture in a comprehensive way (ii) The model is said to be an environmental support system in supporting policy and decision-making in the management	Lamastra et al. [144]

#### Fuzzy Logic Technique in the Food Industry

Fuzzy logic (FL) was first introduced by Zadeh in 1965 based on the impeccable capability of human intellect in decision making and unraveling the imprecise, uncertain, and ambiguous data while solving problems [47, 48]. The fuzzy set theory is recognized in such a manner that an element belongs to a fuzzy set with a certain degree of membership which has a real number in the interval [0, 1] [49]. FL models consist of several steps which are fuzzification, inference system, and defuzzification process [50, 51]. Fuzzification is a process where the crisp value is converted into a degree of membership and yields the fuzzy input sets. The corresponding degree in the membership functions is normally between 0 and 1. [52]. There are a variety of membership functions to choose from, whereby the commonly used ones are triangular, Z-shaped, S-shaped, trapezoidal, and Gaussian-shaped [52]. The inference system is where the fuzzy input is being translated to get output by using the fuzzy rules. The fuzzy rules are known as IF–THEN rules where it is written such IF premise, THEN consequent whereby the IF comprises input parameters and THEN is the output parameters [53]. The inference system consists of the style which is either the Mamdani or Takagi–Sugeno Kang (TSK). Defuzzification is the ultimate phase in the fuzzy logic model where the crisp values are obtained [54]. There are different methods of defuzzification which are center of gravity, mean of maximum, smallest of maximum, largest of maximum, center of maximum, and centroid of area [55].

FL has been long utilized in the industry due to its simplicity and ability to solve problems in a fast and accurate manner. FL has been employed in the food industry in food modeling, control, and classification and in addressing food-related problems by managing human reasoning in linguistic terms [56]. The food manufacturing system has improved by the implementation of the fuzzy logic where about 7% of electricity losses has been reduced compared to the conventional regulation method [57]. Sensory evaluation of the food is also one of the most common parts where FL plays an important role. Furthermore, a quicker solution to problems can be performed by using a system involving fuzzy rules [58]. Table 2 shows previous applications of FL in the food industry and their attributes. From a previous study, FL has been proven to successfully maintain the quality of the foods, and it acts as a prediction tool and control system for food production processes.

**Table 2:** Application of fuzzy logic in the food industry

Application	Objective	Fuzzy inference system	Membership function	Important outcomes	References
Aromatic foods	To rank the sensory attributes of aromatic foods packed in films made from corn starch	Mamdani	Triangular	(i) Overall ranking for tea and tastemaker and the important quality attributes of the food materials in general, and the samples were able to be done using FL (ii) Aroma and taste of tea leaf and tastemaker in general were assessed as “Highly important” sensory attributes	Chowdhury & Das [145]
Beetroot candy	To rank the candy with various content ratios	Mamdani	Triangular	(i) The developed model was able to optimize and perform the ranking of the candy involving different ingredient ratios	Fatma et al. [146]
Canned food	To control sterilization temperature by using fuzzy logic and making online corrections in autoclave operation	Mamdani	Triangular	(i) The sterilizing temperature with an accuracy of ± 0.5 ℃ can be maintained by a fuzzy controller (ii) Batch processing can be completed using the proposed system with less time, steam consumption, and risk of over-sterilization	Chung et al. [147]
Coffee	To determine the suitable process on a dry mill according to customer requirements using an expert system based on fuzzy logic	Mamdani	Triangular	(i) The developed system will be useful for the correct decision process between two different types of coffee (ii) Validation was carried out by comparing the process values by the model with the real process data, and the coefficient of determination obtained was 93%	Hernández-Vera et al. [148]
Coffee beans	To introduce a control system for the roasting machine	Mamdani	Triangular	(i) The consistent roasting level of the beans is able to be produced by the proposed model	Harsawardana et al. [149]
Cupcakes	To rank the cupcakes according to their quality attributes	Mamdani	Triangular	(i) The system was able to determine the best condition for their cupcakes with respect to their sensory attributes (ii) The ranking of the quality attributes was able to be performed by the system	Singh et al. [150]
Dough	To implement the FL to act as a controller system in bread making	Mamdani	Triangular, trapezoidal	(i) The settling time and the response of the FL controller showed a better performance than the proportional-integral-derivative (PID) controller (ii) The FL controller system was established successfully for the proofing process in bread making	Yousefi-Darani et al. [151]
Fava beans	To predict the physical parameters of the beans with various moisture contents	Mamdani	Triangular	(i) The model was able to predict thirteen parameters of the beans with the moisture content ranging from 9.3 to 31.3% in the input (ii) Comparison was done between the FL results and the experimental value where a high correlation value which is 0.999 and mean standard deviation ranging 1.23–12.56% were obtained as an effective system and can be used to develop a model for controlling and managing various stages during processing	Farzaneh et al. (152, 153
Flixweed seed	To rank different ways of extraction in preparing the seeds	Mamdani	Triangular	(i) Ranking based on the properties, extraction output, and duration was able to determine the best method for the preparation of the seeds	Shahidi et al. [154]
Fresh mango juice (litchi juice)	To study the effect of high pressure processes (HPP) on sensory attributes of fresh mango juice and litchi juice	Mamdani	Triangular	(i) The model was able to determine that the HPP effect depended on the type of products and domain of pressure–temperature (ii) HPP is proven to be a promising method for the preservation of fruit products	Kaushik et al. [155]
Hydrogel colloidosomes	To estimate the release of caffeine from hydrogel colloidosome	Mamdani	Type S, type Z	(i) The proposed diffusional-fuzzy model able to describe the caffeine release from hydrogel colloidosomes (ii) The model has a higher precision, better handling of uncertainty property and better generalization capability	Amiryousefi et al. [156]
Onions	To predict the drying kinetics of the onions	Mamdani	Triangular	(i) The model was able to predict the moisture ratio at varying conditions with high performance where the value of R obtained was 0.9999 and the low root mean square error (RMSE) was 0.004157	Jafari et al. [157]
Pineapple Rasgulla	To rank the pineapple Rasgulla with respect to the parameters	TSK	Triangular	(i) Sensory evaluation for different concentrations of pineapple Rasgulla and ranking of the samples was performed successfully by using the FL model	Sarkar et al. [158]
Pizza production industry	To develop a system in order to improve the production system	Mamdani	Triangular	(i) The developed FL control system is able to identify the amounts of workers and ovens needed in pizza production which improves the customer’s satisfactory level by reducing the waiting time as well as reducing the wastage	Blasi [159]
Salt	To estimate the production of salt by variables that affect it	TSK	Triangular	(i) By using the Sugeno zero-order model, the time for production of salt could be estimated with a minor error value of 0.0917	Yulianto et al. [160]
Sardine	To assess fish quality by biogenic amines using a fuzzy logic model	Mamdani	Triangular	(i) The model was able to determine the quality of the fish at the initial stages of storage (ii) The Pearson correlation r value obtained was greater than 0.95 at different temperatures	Zare & Ghazali [161]
Treated raw apple juice	To carry out a sensory evaluation of raw apple juice treated with raw betel leaf essential oil	Mamdani	Triangular	(i) The FL approach by applying similarity analysis gave an insight into variation into customers’ acceptability on treated apple juice	Basak [162]
Wheat dough	To develop an effective sheeting of wheat dough	Mamdani	Trapezoidal	(i) Able to decide the best program with the least number of rolling steps based on the quality of the dough which improves the sheeting process of the dough	Mahadevappa et al. [12]
White mulberry	To forecast the moisture ratio of the mulberry during the drying process	Mamdani	Triangular	(i) The model could predict the moisture ratio of mulberry under varying conditions with a high accuracy where the value of *R*^2^ equals to 0.9996 and RMSE value of 0.01095	Jahedi Rad et al. [163]

##### ANN Technique in the Food Industry

**Fig. 3:**
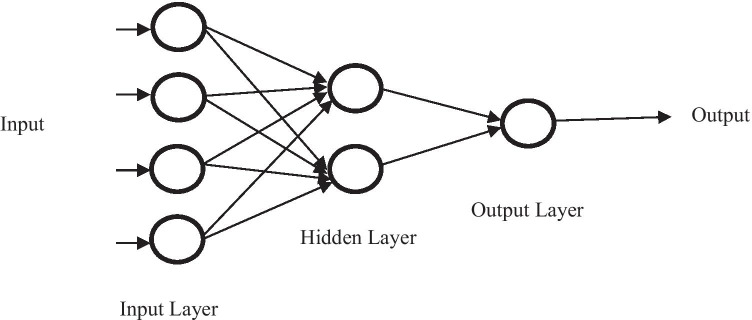
ANN structure in general

**Fig. 4:**
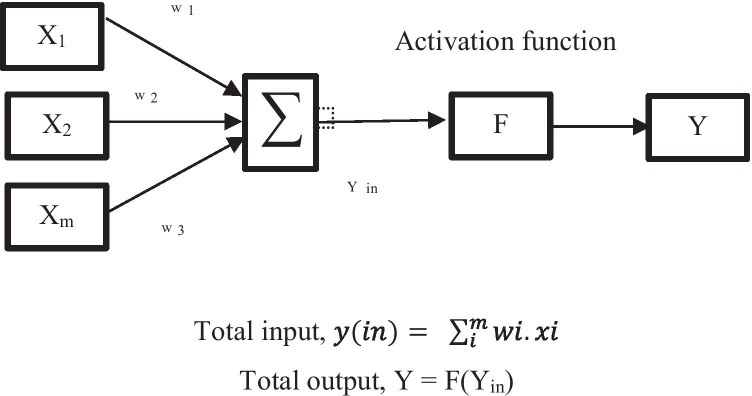
General calculation in ANN

ANN is another AI element, which is also commonly applied in the food industry. ANN is designed to mimic the human brain and be able to gain knowledge through learning and the inter-neuro connections which are known as synaptic weights [59, 60]. Gandhi and coworkers have stated that the configuration of ANN is designed in such a way that it will accommodate certain application such as data classification or pattern recognition [61]. According to Gonzalez-Fernandez, ANN is applicable to a different kind of problems and situations, adaptable, and flexible. In addition, Gonzalez et al. (2019) have also stated that ANN is suitable to model most non-linear systems and is adaptable to new situations even though adjustments are needed. Moreover, the most outstanding features of ANN is its non-linear regression [62]. There are several types of ANN including feedforward neural network, radial basis function neural network, Kohonen self-organizing neural network, recurrent neural network, convolutional neural network, and modular neural network [63]. Multilayer perceptron (MLP), radial basis function networks (RBFNN), and Kohonen self-organizing algorithms are the most effective types of NN when it comes to solving real problems [61]. The most common network that is used for prediction and pattern recognition is the multilayer perceptron [18, 64, 65]. Besides that, ANN learning could be classified into supervised and unsupervised depending on the learning techniques [17]. In general, the structure of ANN consisted of an input layer, hidden layer, and output layer, either single or many layers [66–68]. The architecture comprises activation functions, namely, the feed-forward or feedback [69]. The backpropagation learning algorithm is normally used as it is able to minimize the prediction error by feeding it back as an input until the minimum acceptable error is obtained [18]. An additional input known as bias is added to neurons which allows a portrayal of phenomena having thresholds [70, 71]. In ANN, the dataset is normally associated with a learning algorithm which trained the network and could be categorized into three groups specifically supervised, unsupervised, and reinforcement learning [72]. Then, the data will undergo training and testing for analyzing the outputs. The general structure for the ANN is shown in Fig. 3. The output data can be calculated by using the equation shown based on Fig. 4.

Previous studies have highlighted the utilization of ANN in numerous tasks within the food industry. This includes the assessment and classification of the samples, complex calculation such as heat and mass transfer, and analysis of the existing data for control purposes as well as for prediction purposes which are listed in Table 3. All applications have shown satisfactory performances based on the *R*^2^ values, showing that ANN can provide results in an accurate and reliable manner.

**Table 3:** Application of ANN in the food industry

Application	Objectives	Types of ANN	Outcomes	References
Cocoa powder	To predict the effect of process parameters on the properties of cocoa mixtures	MLP	(i) The model could predict the effect of different parameters changes on the physical and chemical properties of the cocoa mixtures with a high accuracy where the value of *R*^2^ derived was 0.934 with low error of 0.053	Benković et al. [164]
Dried vegetables	To identify the quality of dried carrots and classification of such dried products	MLP	(i) Suitable to be used for assessment and classification of dry carrots (ii) Enable the selection of important representative characteristics for the quality assessment (iii) Comparison between algorithms was done, and the backpropagation is the most successful algorithm compared to other algorithms	Koszela et al. [165]
Eggplant	To describe the mass transfer kinetics of eggplants in osmotic dehydration (OD)	MLP	(i) High volume of complicated problems is able to be modeled and analyzed by using ANN, and it is the most suitable software for calculation problems (ii) The developed model achieved the highest *R*^2^ value of 0.9825	Bahmani et al. [166]
Extra virgin olive oils	To evaluate the influence of light exposure conditions and packaging material on the stability of physicochemical characteristics of extra virgin olive oils	MLP	(i) ANN showed a high classification performance with an accuracy of greater than 90% for the test data and greater than 85% for the training set (ii) Showed the robustness of the model which indicates the suitability for solving clustering, pattern recognition, classification, and adulteration issues regarding extra virgin olive oils	S. F. Silva et al. (71, 70
Garlic	To forecast the sensory quality of garlic	MLP	(i) A model with the best prediction of the sensory quality of the garlic and garlic products was developed with an *R*^2^ value of 0.9866	Liu et al. [167]
Honey	To predict the stability of the Indian honey crystallization of different component ratios	MLP	(i) The ANN model was able to forecast the stability of the honey consisting of different compositions with high accuracy where the *R* value obtained was 0.9994	Naik et al. [168]
Mango	To estimate the weight of the mango	MLP	(i) Estimation of the weight of the mangoes was able to be done by applying thirteen different parameters in the model	Dang et al. [169]
Mushrooms	To develop an ANN model that can predict the moisture content of the mushrooms during the drying process	MLP	(i) The ANN model was able to predict the moisture content of the mushrooms during the drying process with an *R* value of 0.9817	Omari et al. [170]
Mushrooms	To predict the temperature variety of mushroom growing hall based on the parameters affecting the room temperature	MLP, RBF	(i) The prediction by using the RBF method has a higher accuracy compared to that of MLP where the value of correlation achieved was 0.996 and 0.9612, respectively	Ardabili et al. [171]
Onions	To estimate the drying behavior of onion	MLP	(i) The develop model was able to forecast the drying kinetics of onions at different temperatures and times with a high performance where the *R* value achieved a value of 0.99956	Jafari et al. [157]
Potato cubes	To carry out analysis in a fluidized bed dryer for the drying of potato cubes under different conditions	MLP	(i) The developed model was able to carry out the analysis of energy and exergy losses in the dryer for the drying of the potato cubes (ii) All the analysis done by using the ANN model obtained a high value of *R*^2^ which is greater than 0.98 and the average value obtained was 0.99	Azadbakht et al. [172]
Potato peels	To model a system that can predict and optimize the extracted conditions by using the response surface methodology and ANN	MLP	(i) This study with the aid of the ANN model actually helped to determine that the potato peels which are often thrown away as a waste are actually beneficial (ii) The *R*^2^ value achieved by the ANN model for the analysis of three different extracted values were greater than 0.93	Anastácio et al. [173]
Quince fruit	To determine the moisture ratio of the fruit during the drying process and test the performance of the developed model	MLP	(i) By implementing different ANN structures, the moisture content ratio during the drying process for the quince fruits was modeled successfully (ii) The developed model was able to predict the moisture ratio with high correlation value where the *R*^2^ obtained was greater than 99%	Chasiotis et al. [174]
Rice crop	To predict rice production yield and investigate the factors affecting the rice crop yield	MLP	(i) A good alternative to predict the rice production yield compared to traditional linear regression methods (ii) The accuracy obtained by the developed algorithm was 97.54%, with the sensitivity of 96.33% and specificity of 98.12%	Gandhi et al. [61]
Sausage	To forecast the benzo[a]pyrene (BaP) content of smoked sausage	MLP	(i) The model was able to predict the BaP content in smoked sausages and create a control system for the smoking to reduce the BaP contamination in smoked sausages (ii) The model has a high accuracy where the overall prediction was greater than 0.90	Chen et al. [175]
Vegetable oils	To classify vegetable oils: canola, sunflower, corn, and soybean using a very few mathematical manipulation and ANN	MLP	(i) Able to simplify the vegetable oil classification with high accuracy (ii) A fast-network training and uses very few mathematical manipulations in the spectra data	Silva et al. [71], Da et al. [70]

#### Machine Learning Techniques

Machine learning (ML) is known to be the subset of AI [73, 74]. It is a computer algorithm that advances automatically with experiences. ML can be classified into three broad categories which are supervised learning, unsupervised learning, and reinforcement learning [11, 75]. Supervised learning aims to predict the desired target or output by applying the given set of inputs [76]. On the other hand, unsupervised learning does not have any outputs to be predicted and this method is utilized to classify the given data and determine the naturally occurring patterns [77]. Reinforcement learning is when there is an interaction between the program and the environment in reaching certain goals [78]. Among the known models in machine learning are ANN, decision trees (DT), support vector machines (SVM), regression analysis, Bayesian networks, genetic algorithm, kernel machines, and federated learning [76, 79]. ML has been commonly used for handling complex tasks and huge amount of data as well as variety of variables where no pre-formula or existing formula is available for the problem. Other than that, ML models have the additional ability to learn from examples instead of being programmed with rules [80].

Among the ML methods that are used in the food industry include ordinary least square regression (OLS-R), stepwise linear regression (SL-R), principal component regression (PC-R), partial least square regression (PLS-R), support vector regression (SVM-R), boosted logistic regression (BLR), random forest regression (RF-R), and k-nearest neighbors’ regression (kNN-R) [81]. Studies showed that the usage of ML has helped in reducing the sensory evaluation cost, in decision making, and in enhancing business strategies so as to cater users’ need [82]. Long short-term memory (LSTM) which is an artificial recurrent neural network has been employed in the food industry as pH detection in the cheese fermentation process [83]. On the other hand, GA has been utilized for finding the optimum parameters in food whereas NN has been occupied to predict the final fouling rate in food processing [84]. ML has shown to be advantageous in predicting the food insecurity in the UK [85]. Apart from that, ML has also proven to have predicted the trend of sales in the food industry [86] In addition to that, ML was also able to predict the food waste generated and give an insight to the production system [87]. Major applications of ML in the food industry and its positive highlights are briefly emphasized in Table 4.

**Table 4:** Application of machine learning in food industries

Application	ML methods	Important outcomes	References
Apple	Linear discriminant analysis, adaptive boosting	(i) The ML was able to classify the apples accurately with a rate of 100% using the collected acoustic emission signals	Li et al. [176]
Artichoke	MLP, RF, BLR	(i) The characteristics of ion patterns were done by the ML models to be set up for each enzyme with a high prediction rate of 95% above	Sabater et al. [177]
Beer	ANN	(i) The ANN model was able to classify the chemical components in the beer with a high overall accuracy of 95%	Claudia Gonzalez et al. [178]
Biscuits	Convolutional neural network	(i) The developed model was able to classify and evaluate the quality of different types of biscuits with an accuracy up to 99%	De Sousa Silva et al. [179]
Cheese	LSTM	(i) The combination of mechanistic modeling with LSTM was able to describe the changes in lactic acid, lactose, and biomass with high accuracy where the value of *R*^2^ obtained is greater than 0.99 (ii) The prediction of pH for the cheese fermentation is able to be done by using the developed model	Li et al. [83]
*Citrus limetta* (Mosambi peel)	SVM-ANN, SVM-Gaussian process regression (SVM-GPR)	(i) The SVM model was able to predict and classify all the results for the taste of lime powder that has been treated by the salt with an accuracy of 1.0 (ii) Optimization by using the ML tool allows to maintain the taste and retain the polyphenol content in the lime	Younis et al. [180]
Fruits (*Arbutus unedo L.* fruits)	RF, SVM, ANN	(i) The stability of the extracts in the *Arbutus unedo L.* fruits in terms of aqueous and powder systems was able to be done by the ML methods with overall coefficient in the range of 0.9128 and 0.9912 for the best models chosen	Astray et al. [181]
Lamb meat	SVM	(i) The classification accuracy of the lamb meat fat increased from 89.70 to 93.89% by using the SVM method	Alaiz-Rodriguez & Parnell [85]
Mangoes	Naive Bayes, SVM	(i) The system is able to detect the maturity of the mangoes based on their quality attributes	Pise & Upadhye [182]
Meat	OLS-R, SL-R, PC-R, PLS-R, SVM-R, RF-R, and kNN-R	(i) Different kinds of microorganisms causing the beef spoilage could be detected by using the regression ML that obtained the data from five different analytical methods (ii) All the methods were able to predict in all cases accurately with the rank order of RF-R, PLS-R, kNN-R, PC-R, and SVM-R	Estelles-Lopez et al. [81]
Milk	SVM	(i) Presence and the level of antibiotics concentration in the cow milk was determined by using the SVM classifiers with high accuracy rate of greater than 83% and greater sensitivity compared to the typical metrics	Gutiérrez et al. [183]
Salmon	TreeBagger	(i) The established model was able to classify the normal and freezer burnt categories with high accuracy where the correct classification rate yielded 0.914 for validation and 0.978 in cross validation	Xu & Sun [184]
Wine	SVM, RF, MLP	(i) The comparison among the three algorithms were done in the evaluation of wine quality and the best result was obtained by the RF method with an average accuracy of 81.96% meanwhile others delivered a low accuracy late. This indicates the RF algorithm can be used to evaluate the quality of the wine	Shaw et al. [185]

#### Adaptive Neuro Fuzzy Inference System (ANFIS) Techniques

**Fig. 5:**
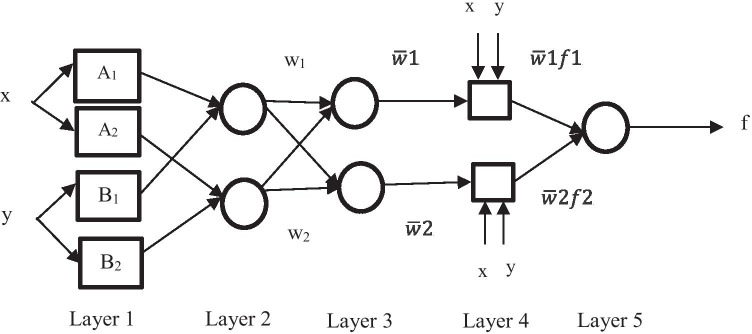
General structure of ANFIS

ANFIS is a type of AI where FL and ANN are combined in such a way that it integrates the human-like reasoning style of the FL system with the computational and learning capabilities of ANN [56]. In ANFIS, the learning procedure is transferred from the neural network into the FL system where a set of fuzzy rules with suitable membership functions from the data obtained is developed [88]. Mamat et al. [89] stated that uncertainty data could be processed and gain higher accuracy when ANFIS is applied [89]. Besides, ANFIS is also known as a fast and robust method in solving problems [90]. Not only that, Sharma et al. [91] also claimed that ANFIS has a higher performance compared to other models such as ANN and multiple regression models in their study [91]. ANFIS is a fuzzy reasoning system and combination of the parameters trained by ANN-based algorithms. The fuzzy inference system that is normally used is Takagi Sugeno Kang in the ANFIS model with the feedforward neural network consisting of the learning algorithms [92]. The structure of ANFIS is made up of five layers which are fuzzy layer, product layer, normalized layer, defuzzification layer, and total output layer [93, 31, 32]. The backpropagation algorithm has been normally applied in the model in order to avoid over-fitting from occurring [92]. A high correlation value (*R*^2^) indicates that the developed model has high accuracy and is suitable for industrial applications. The general structure of the ANFIS model is illustrated in Fig. 5.

The first layer in ANFIS has nodes that are adjustable, and it is called as the premise parameters [56]. The second layer in ANFIS has fixed nodes, and the output is the product of all incoming signals. Every output node represents the firing strength of the rule. The third layer consists of fixed node labeled as N. The outputs of the third layer are called normalized firing strengths. Every node in the fourth layer is an adaptive node with a node function, and the parameters in this layer are called as the subsequent parameters [56]. The final layer in the ANFIS layer has a fixed single node which calculates the overall output as the summation of all the incoming signals. The calculation involved in each layer is shown below. The output of the ith model in layer 1 is denoted as 0_1_, i.

Layer 1: $${O}_{1,i}= {\mu }_{Ai}\left(x\right), for i=\mathrm{1,2}$$ atau $${O}_{1,i}= {\mu }_{Bi-2}\left(y\right), for i=\mathrm{3,4}$$.

Layer 2: $${O}_{2,i}= {w}_{i}={\mu }_{Ai}\left(x\right){\mu }_{Bi}\left(y\right), for i=\mathrm{1,2}$$.

Layer 3: $${O}_{3,i}=\overline{w }= \frac{{w}_{i}}{{w}_{1}+{w}_{2}}, i=\mathrm{1,2}$$.

Layer 4: ${O}_{4,i}= \overline{w}{f }_{i}={\overline{w} }_{i}({p}_{i}x+{q}_{i}y+{r}_{i}$); ${w}_{i}$ is the normalized firing strength from layer 3 and.

{${p}_{i},{q}_{i},{r}_{i}$} is the parameter set of this node.

Layer 5: $${O}_{\mathrm{5,1}}=\sum_{i}{\overline{w} }_{i}{f}_{i}= \frac{\sum_{i}{w}_{i}{f}_{i}}{\sum_{i}{w}_{i}}$$.

The ANFIS model is attractive enough that it could solve problems related to the food industry, which are complicated, practical, and barely solved by other methods and has been widely used in the food industry for prediction and classification purposes. ANFIS has been applied in various food processing involving recent technology which comprised five main categories which are food property prediction, drying of food, thermal process modeling, microbial growth, and quality control of food as well as food rheology [56]. The utilization of ANFIS in the food industry has been commenced years ago, and Table 5 describes those applications.

**Table 5:** Application of ANFIS in the food industry

Applications	Outcomes	References
Fish oil	(i) A model was developed to estimate the oxidation parameters using three different algorithms which are, ANFIS, multilinear perceptron, and multiple linear regression, and it was found that ANFIS model had the best accuracy in predicting the parameters	Asnaashari et al. [186]
Ice cream	(i) The sensory attributes of ice cream were investigated by using the ANFIS model to predict the acceptability of taste with respect to the input parameters and the model achieved a minimum error of 5.11% and high correlation value of 0.93	Bahram-Parvar et al. [187]
Indian sweets (*Pantoa*)	(i) The prediction of the heat transfer coefficient during the frying of *pantoa* using the ANFIS model yielded a high *R*^2^ value of 0.9984, and this prediction is important for designing the process equipment as well as saving energy in commercial production	Neethu et al. [188]
Orange	(i) The developed ANFIS model was able to predict the orange taste and has higher performance when compared to multiple regression model	Mokarram et al. [189]
Quince fruits	(i) The ANFIS model was able to predict the moisture ratio, energy utilization, energy utilization ratio, exergy loss, and exergy efficiency of quince fruit during the drying process with high accuracy of with *R*^2^ value of 0.9997, 0.9989, 0.9988,0.9986, and 0.9978, respectively (ii) The ANFIS model was compared with the ANN model, and the results obtained showed that the ANFIS model has higher accuracy compared to the ANN model where the value of *R*^2^ was higher with a lower error value in the ANFIS model	Abbaspour-Gilandeh et al. [190]
Rapeseed oil	(i) The developed ANFIS could predict the different outputs of rapeseed oil process by oil extraction and cooking at industrial scale, and the model achieved a high correlation coefficient which is around 0.99	Farzaneh et al. [152, 153]
*Salmonella enteritidis*	(i) Prediction of the inactivation of *Salmonella enteritidis* by ultrasound was able to be done by the developed ANFIS model with a good accuracy where the correlation coefficient obtained was 0.974 (ii) This study was known to be important in the food industry as the bacteria can cause food poisoning if proper detection is not being done	Soleimanzadeh et al. [191]
Taro	(i) The optimization of extraction conditions of antioxidants from the taro flour can be done by using the developed ANFIS model coupled with response surface methodology (ii) The prediction values obtained from the developed model were validated by comparing with the experimental values, and the results were almost consistent with prediction values from the developed model	Kumar & Sharma [192]
Vegetables (cantaloupe, garlic, potatoes)	(i) The developed model by using the ANFIS system was able to predict the effective moisture diffusivity, specific energy consumption, moisture ratio, and drying rate of the vegetables with a high regression coefficient of 0.9990, 0.9917, 0.9974, and 0.9901, respectively, with minimum error value (ii) Comparison between the ANFIS model and ANN model was carried out, and the results showed that the ANFIS model possess a higher efficiency than that of ANN model	Kaveh et al. [193]
Virgin olive oil	(i) The ANFIS model was able to predict the quality of virgin olive oil samples with high accuracy where the coefficient determination obtained was greater than 0.998 (ii) It was also able to visualize the effects of temperature, time, total polyphenol, fatty acid profile, and tocopherol on the oxidative stability of virgin olive oil	Arabameri et al. [194]
Yam	(i) The prediction of the yam moisture ratio during the drying process showed a high *R*^2^ value with 0.98226 by using the developed ANFIS model	Ojediran et al. [195]

#### Integrating AI with External Sensors for Real-time Detection in Food Industry

FL or ANN is often integrated with several sensors for real-time detection such as electronic nose (E-nose), electronic tongue (E-tongue), machine learning (ML), computer vision system (CVS), and near infrared spectroscopy (NIRS) for real-time detection and to obtain higher accuracy results in a shorter time. These detectors have also combined their elements together for enhancing their accuracy and targeted results. The integration of these sensors with the artificial intelligence methods has been shown quite a number in food industries over the past few years.

**Fig. 6:**
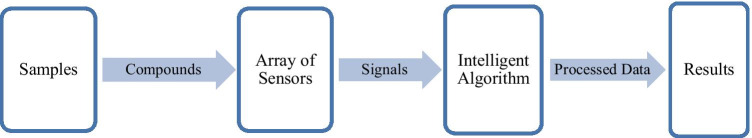
E-nose system

Electronic nose also known as E-nose is an instrument created to sense odors or flavors in analogy to the human nose. It consists of an array of electronic chemical sensors where it is able to recognize both simple and complex odors [94]. E-nose has been used in gas sensing where the analysis of each component or mixture of gases/vapors is required. Besides, it plays an important role in the food industry for controlling the quality of the products. Due to its ability to detect complex odors, it has been employed as an environment protection tool and detection of explosives materials [95]. An array of non-specific gas sensors is known to be the main hardware component of E-nose where the sensors will interact with a variety of chemicals with differing strengths. It then stimulates the sensors in the array where characteristic response is extracted known as a fingerprint [94]. The main software component of E-nose is its feature extraction and pattern recognition algorithms where the response is processed, important details are elicited and then chosen. Thus, the software component of the E-nose is greatly important to stimulate its performance. In general, E-nose is divided into three main parts, namely, sample delivery system, a detection system, and a computing system. ANN, FL, and pattern recognitions are the examples of the methodology employed in E-nose [96]. The general system of E-nose is shown in Fig. 6.

E-nose has been widely used to aid in both quality control and assurance in the food industries. Wines, grains, cooking oils, eggs, dairy products, meat and dairy products, meat, fish products, fresh-cut and processed vegetables, tea, coffee, and juices have successfully applied e-nose for sampling classification, detection, and quality control. E-nose has successfully classified samples with different molecular compounds [97]. Besides, Sanaeifar et al. have reviewed and confirmed that e-nose was able to detect defects and contamination in foodstuffs [98]. Classification and differentiation of different fruits have also determined by using e-nose [99]. A review has been conducted on the application of the E-nose for monitoring the authenticity of food [100]. Adding to this, Mohamed et al. have carried out a comprehensive review on the classification of food freshness by using e-nose integrated with the FL and ANN method [101]. Recent application of e-nose with computing methods involving AI in food industries is shown in Table 6.

**Table 6:** Application of E-nose with AI in food industries

Application	Objectives	AI technique	Outcomes/impacts	References
Beef	To classify the beef samples	Adaptive FL system (AFLS); ANFIS	(i) The accuracy of the AFLS model was very good which was 94.28% for overall correct classification which shows that it is able to tackle complex, non-linear problems like meat spoilage (ii) By using the ANFIS method, the results were satisfactory but were obtained with high computational cost (iii) E-nose with appropriate machine learning tools proved that it is useful to monitor the spoilage of the meat during aerobic stage at various temperatures	Kodogiannis & Alshejari [196]
Cocoa	To figure out and classify the fermentation time of the cocoa beans	DT, boosted tree RF, ANN, KNN, naïve Baiyes (NB)	(i) The ANN and Boosted tree algorithm manage to obtain an acceptable classification rate while the fermentation time was not able to be determined by the KNN and NB algorithm	Tan et al. [197]
Coffee beans	To forecast the level of acidity in fresh roasted beans	ANN	(i) The model was able to predict the acidity level values with an accuracy around 95% based on human sourness level scores	Thazin et al. [198]
Chicken meat	To classify the fresh and freeze-thawed chicken meat	Fuzzy K-nearest neighbors algorithm (FK-NN)	(i) The FK-NN algorithm showed a high performance, and it can be used in e-nose to identify and classify the fresh and frozen-thawed chicken meat	Mirzaee-Ghaleh et al. [199]
Cow ghee	To detect the adulteration of the margarine in cow ghee	ANN	(i) The ANN model was able to analyze the data obtained from the e-nose with high accuracy	Ayari et al. [200]
Edible oil	To detect the adulteration in oxidized and non-oxidized edible oil	ANN	(i) The developed ANN model with e-nose was able to detect the adulteration in the edible oil with high accuracy (ii) The classification of the system was compared with other methods and it was given that ANN had the highest classification rate with 97.3%	Karami et al. [201]
Fish	To identify and classify the fish spoilage	ANN, PCA	(i) The developed model using the PCA and ANN was able to classify the fish according to their spoilage group with an accuracy of 96.87%	Vajdi et al. [202]
Fruits	To apply Kernel extreme learning machines in E-nose for recognition and perception of fruit odors	Kernel extreme machine learning	(i) The proposed system performed significantly good in odor recognition (ii) It achieved higher testing accuracy and smallest value of training time and testing time compared to other systems that were used for the comparison for recognizing the fruit odors	Uçar & Özalp [203]
Fruits	To apply artificial bee colony (ABC) algorithm in ANN to classify data from electronic nose and evaluate its performance	ANN & artificial bee colony	(i) ABC-ANN is more successful in odor classification of e-nose data and has higher performance compared to the backpropagation algorithm that was used (ii) The system was able to classify four different aroma which are the aroma of the lemon, cherry, strawberry, and melon	Adak & Yumusak [204]
Fruit juice	To figure out the amount of the food additives in the fruit juice	RF, SVM, ELM, PLSR	(i) The additives which are benzoic acid and chitosan in the juice could be predicted with accurately by using the ELM and RF methods with *R*^2^ value of 0.92 and 0.91, respectively (ii) ELM and RF method has a higher accuracy in predicting the additives when compared to ELM and PLSR where the *R*^2^ value obtained by ELM and PLSR are 0.72 and 0.51, respectively	Qiu & Wang [205]
Honey	To determine the best classifier in forecasting the physicochemical properties of Iranian ziziphus honey samples	ANN, SVM	(i) Both the ANN and SVM model combined with the e-nose showed a good prediction rate in determining the physicochemical properties of the honey sample (ii) The developed model using ANN method shower a higher accuracy in the prediction compared to that of with SVM method	Faal et al. [206]
Lemon	To characterize and predict the quality of various lemon slices	ELM, RF, SVM	(i) Prediction using the ELM method obtained the highest accuracy of 0.959 followed by RF and SVM with 0.935 and 0.922 respectively which indicates that ELM integrated with the E-nose is the best classification model	Guo et al. [207]
Pear	To enhance the food quality by optimize the drying process of balsam pear	FL	(i) The drying time was able to be shortened to retain the aroma and enhance the product quality (ii) Designation of industrialized control method was established to simplify the control and has good drying effects	Li et al. [208]
Pork meat	To differentiate between the fresh and frozen-thawed meat	BPANN	(i) Combined ANN with E-nose was able to distinguish three types of meat which are loin, neck and ham (ii) The model was able to differentiate the fresh meat from spoiled meat and frozen meat with overall sensitivity of 85.1% and 97.5% specificity	Górska-Horczyczak et al. [209]
Rice grains	To detect the *Sitophilus oryzae* infestation in stored rice grains	FL ARTMAP PCA, MLR	(i) E-nose with the application of FL ARTMAP is useful when the data are exhaustive and deductions about analysis are needed to be done (ii) The model was able to classify the grains of infested, non-infested, required treatment and others (iii) The hybrid system is beneficial in the food and grain industry where early detection of infestation in grain can be done to minimize the post-harvest losses	Srivastava et al. [210]
Rice grains	To classify the *Rhyzopertha. Dominica* infested rice grains and recognized them	ANN	(i) BPANN with e-nose system gave the highest *R*^2^ value which is 0.98 compared to other methods that were used with the e-nose for the classification process and it has the highest accuracy compared to the rest (ii) The system was able to predict the infested rice grains for different days	Srivastava et al. [211]
Shelled peanuts	To assess the storage quality of shelled peanuts	FL	(i) The FL analysis able to screen and rank the e-nose sensors and the discard time for the shelled peanuts was able to be determined (ii) The storage time prediction of shelled peanuts using sensors closely matched with the conventional methods (iii) It can be an eco-friendly alternative as it is rapid and not destructive	Raigar et al. [212]
Salmon	To classify the level of freshness of the salmon samples	PCA, CNN, SVM	(i) The developed system was able to detect, cluster, and classify the salmon according to their freshness level, and the overall accuracy obtained by the CNN-SVM model was greater than 90%	Feng et al. [213]
Saffron	To detect the adulteration of saffron samples	ANN	(i) The classification of original and adulterated saffron was successfully done by using e-nose with the pattern recognition method with 100 and 86.87% accuracy, respectively	Heidarbeigi et al. [214]
Saffron	To identify different aromas of Iranian saffron	ES, PLS, ANN	(i) E-nose coupled with ANN successfully obtained 100% for the classification of saffron samples	Kiani et al. [215]
Spinach	To detect the postharvest freshness of spinach	ANN	(i) Combination of E-nose with the BPNN method proves to be effective for a fast and non-destructive way to detect the spinach freshness with an accuracy of 93.75% for the classification accuracy	Huang et al. [216, 231]
Strawberry juice	To classify and carry out analysis by using E-nose with neural networks and other learning methods as well to evaluate the performance of the system	ANN, ML	(i) E-nose could differentiate each treatment type and be able to predict the quality parameters (ii) ELM network was able to classify the strawberry juice and is much faster compared to other modelling networks that were used (iii) ELM also showed a better performance compared to other modelling techniques that were being used	Qiu et al. [217]
Sunflower oil (SO)	To determine the frying disposal point of SO	FL	(i) E-nose combined with FL was able to assess the frying disposal time of fried SO blend (ii) The combination of e-nose with FL has potential for other fried product platforms	Upadhyay et al. [218]
Wheat grain	To determine the granary weevil infestation in stored wheat grains	FL	(i) The most responsive sensors and specific VOCs generated by insect-infected wheat grains were able to be screened out by the e-noses sensor associated with the fuzzy logic analysis (ii) E-nose was proven to be a potential method for accurate and rapid in monitoring the infestation in stored wheat grain. It is also a reliable method for industries to determine the quality of the product throughout the storage period	Mishra et al. [219]

**Fig. 7:**
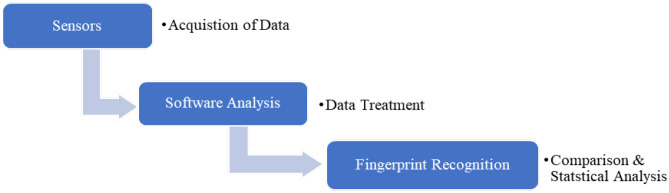
E-tongue system

Electronic tongue (E-tongue) is an instrument that is able to determine and analyze taste. Several low-selective sensors are available in E-tongue which is also known as “a multisensory system,” and advanced mathematical technique is being used to process the signal based on pattern recognition (PARC) and multivariate data analysis [102]. For example, different types of chemical substances in the liquid phase samples can be segregated using E-tongue. About seven sensors of electronic instruments are equipped in E-tongue, which enabled it to identify the organic and inorganic compounds. A unique fingerprint is formed from the combination of all sensors that has a spectrum of reactions that differ from one another. The statistical software of E-tongue enables the recognition and the perception of the taste. E-tongue comprises three elements specifically the sample-dispensing chamber or automatic sample dispenser, an array of sensors of different selectivity, and image recognition system for data processing (Ekezie, 2015). Samples in liquid forms could be analyzed directly without any preparation while the samples in solid forms have to undergo preliminary dissolution before measurement is carried out. The process of E-tongue system is shown in Fig. 7 below. The ability to sense any taste like a human olfactory system makes it one of the important devices in the food industry, especially for quality control and assurance of food and beverages [103]. In addition, E-tongue has been used to identify the aging of flavor in beverages [104], identify the umami taste in the mushrooms [105], and assess the bitterness of drinks or dissolved compounds [102]. Jiang et al. performed a summarized review on the application of e-nose in the sensory and safety index detection of foods [106]. Moreover, the demand of E-tongue in the food industry market has risen due to the awareness on delivering safe and higher-quality products. The details of recent applications of E-tongue in the food industry are shown in Table 7.

**Table 7:** Application of E-tongue with AI in food industries

Application	Objectives	AI technique	Outcomes/impacts	References
Ham	To monitor the salt processing of hams salted differently with different formulations	Simplified fuzzy ARTMAP neural network	(i) The data obtained from the e-tongue was able to be analyzed and classified using ANN (ii) The data was classified using two processing variables which are the processing time and salt formulation (iii) Optimum parameters value for SFAM neural networks were drawn out to be used in the microcontroller device	Gil-Sánchez et al. [220]
Honey	To differentiate different types of honey according to their antioxidant level	Fuzzy ARTMAP neural network (FAM)	(i) The proposed E-tongue system was able to differentiate different types of honey as well as their total antioxidant capacity level (ii) The ANN fuzzy art map type analysis had a high classification success rate of 100% which indicates that it is a good measurement system	Marisol et al. [221]
Liquor	To classify different types of Chinese liquor flavor using e-tongue with fuzzy evaluation and prediction by SVM	SVM & fuzzy evaluation	(i) E-nose with the SVM system was able to classify four different flavors of liquor with an accuracy of 100% (ii) The developed system is able to discriminate the samples accurately and the output evaluation language in line with the human perception	Jingjing et al. [222]
Milk	To detect the adulteration of raw milk	SVM	(i) The developed model was able to determine the adulteration in the samples with a high accuracy values which are all greater than 87% for different types of adulterants in the milk	Tohidi et al. [223]
Peanut meal	To assess the taste attributes of peanut and compare the predictive abilities of the methods used	ANN, partial least square (PLS)	(i) Good stability and repeatability with respect to the measured signals were exhibited by the sensors in the E-tongue (ii) Different concentrations with the same taste (five types of taste) were able to be discriminated by the E-tongue (iii) RBFNN has a better prediction ability with lower error and higher correlation coefficients than those of the PLS method	Wang et al. [224, 225]
Pineapple	To classify the pineapples according to their sweetness level and determine the best algorithm	SVM, KNN, ANN, RF	(i) Different machine learning algorithms were employed in determining the sweetness of the pineapple, and the best algorithm obtained was the KNN method where it achieved an accuracy of 0.820 (ii) The developed model will be beneficial in industry when the selection of pineapples in large quantities is required	Hasan et al. [226]
Rice	To discriminate and predict the solid foods as well as to provide an assessment tool for food industries	ANN	(i) RBFNN was able to distinguish different types of rice with 95% accuracy in classification (ii) Voltametric E-tongue is useful for the qualitative analysis for rice	Wang et al. [224, 225]
Rice	To develop similarity analysis combined with artificial neural networks (SA-ANN) in e-tongue for the prediction of rice sensory quality	ANN	(i) SA-ANN in E-tongue was able to predict the rice sensory quality and carry out systematic analysis (ii) Comparison was carried out between PCA-ANN and SA-ANN, and it was found that SA-ANN has better precision and accuracy compared to PCA-ANN (iii) SA-ANN is a less-labor intensive, quicker method and has potential for rapid and big scale prediction of rice sensory property	Lu et al. [227]
Sugarcane	To characterize and apply voltametric e-tongue for the analysis of glucose from the sugarcane	ANN	(i) Multilayer ANN with wavelet information was able to process complex responses from the E-tongue (ii) The proposed model is suitable to be used for hydrolyzed samples from sugarcane bases	De Sá et al. [228]
Tangerine peel	To classify tangerine peel of different ages	BPNN, ELM	(i) The model was able to classify the tangerine peel samples of different ages (ii) Comparison was done for few linear models and non-linear models, and it was obtained that non-linear models exhibited better performance than linear models (iii) ELM was the best for the classification of the samples with high accuracy followed by BPNN	Shi et al. [229, 230]
Teas	To distinguish different types of teas	ANN, SVM	(i) Different types of teas were able to be distinguished by using the developed system and the compositions of the tea also could be identified	Huang et al. [216, 231]
Tilapia fillets	To predict the changes in freshness of tilapia fillets at different temperatures using the combined techniques	ANN-PCA	(i) E-tongue is able to distinguish the extracts of tilapia fillets stored at different days and different temperatures (ii) The model set up is able to predict the freshness of tilapia fillets stored at different temperatures ranging from 0 to 10 °C	Shi et al. [229, 230]

**Fig. 8:**
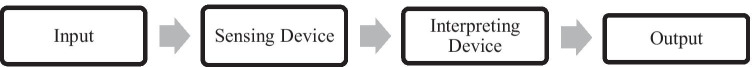
Working Principle of CVS

**Fig. 9:**
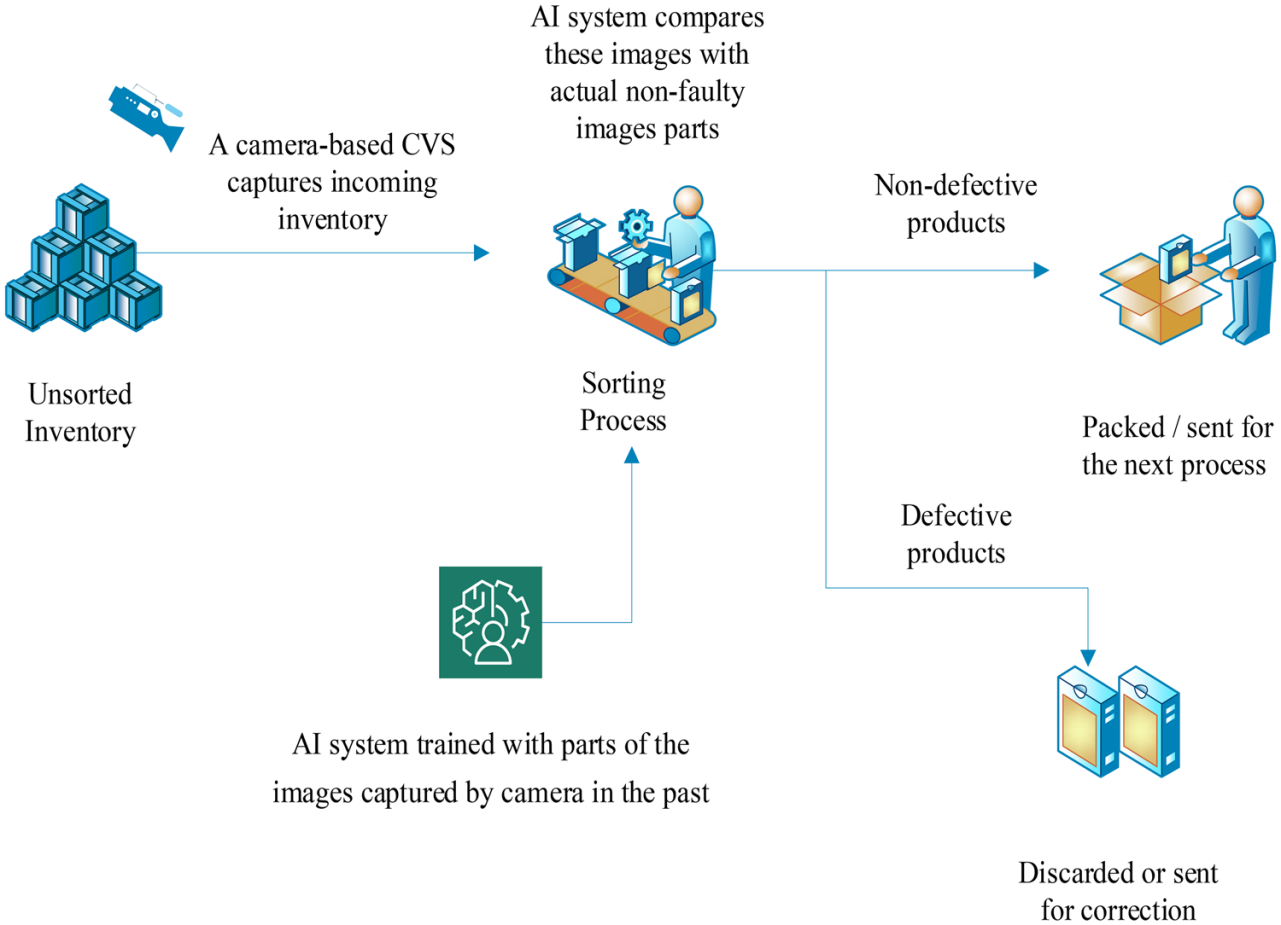
CVS-based quality control process

The computer vision system (CVS) is a branch of AI that combines the image processing and pattern recognition techniques. It is a non-destructive method that allows the examination and extraction of image’s features to facilitate and design the classification pattern [107]. It is also recognized as a useful tool in extracting the external feature measurement such as the size, shape, color, and defects. In general, it comprised a digital camera, a lighting system, and a software to process the images and carry out the analysis [108]. The system can be divided into two types which are 2D and 3D versions. Its usage is not restricted to various applications in food industries such as evaluating the stages of ripeness in apples [107], predicting the color attributes of the pork loin [109], detecting the roasting degree of the coffee [110], evaluating the quality of table grapes [111], and detecting the defects in the pork [112]. The combination of CVS with soft computing techniques has been said as a valuable and important tool in the food industry. This is because the combination of these systems offers good advantages such as an accurate prediction in a fast manner can be achieved. Table 8 shows the combination of CVS and soft computing that has been used in the food industry. Figure 8 shows the working principle of CVS. An example on the utilization of CVS for the quality control is shown in Fig. 9 [113].

**Table 8:** Application of CVS with AI in food industries

Application	Objectives	AI technique	Important outcomes	References
Apple	To sort the defective and normal apples	CNN, SVM	(i) The developed CNN with the CVS model was able to classify the apples with a high accuracy rate of 96.5%, and it was proven to be more effective than the conventional image processing method which was combined with the SVM classifier where the accuracy rate was 87.1%	Fan et al. [232]
Apple slices	To study the drying effects on the changing color of the apple slices	ANN	(i) CVS was able to track the color changes during the drying process, and the combination with the ANN was able to estimate the quality of the apple during the drying process ii) The developed model achieved *R*^2^ values of greater than 0.92 for all the analyses	Nadian et al. [233]
Banana	To classify the banana according to its ripeness	ANN, SVM, KNN, DT	(i) The ANN-based model system has a higher classification rate compared to the other algorithms with the highest overall recognition rate of 97.75%	Mazen & Nashat [234]
Barley flour	To predict the barley flour based on the improvised method	SVM, KNN, DT, RF	(i) The developed model of CVS with different learning algorithms was improvised by using the spatial pyramid partition ensemble method for the classification of the barley flour where the accuracy achieved was 75% (KNN), 95% (SVM & RF), and 100% (DT)	Lopes et al. [235]
Beer	To forecast the beer acceptability based on different sensory parameters	ANN, ML	(i) Seventeen ML algorithms were used to find the best model with a good performance was carried out, and the results showed that Bayesian regularization had the best accuracy where the *R* value obtained was 0.92 (ii) The combination of RoboBEER, CVS, and ANN algorithms allowed to determine the beer making based on its acceptability of customers and its quality	Gonzalez Viejo et al. [236]
Bell pepper	To describe the ripeness level of bell pepper automatically	ANN, FL	(i) An artificial vision system was able to be developed by using the CVS and ANN/FL in predicting the maturity of the bell pepper (ii) The model using RBF-ANN has a higher classification accuracy compared to FL where the maximum accuracy obtained by both models are 100% and 88%, respectively	Villaseñor-Aguilar et al. [237]
Cape gooseberry	To classify the ripeness of cape gooseberry	ANN, SVM, DT, KNN	(i) All the models were able to classify the ripeness of the cape gooseberry with a high accuracy where the accuracy obtained by all the models was greater than 86% using different color spaces, which indicates that it is a good classifier system	Castro et al. [238]
Coffee beans	To develop a system that can classify the coffee beans	ANN	(i) Green coffee beans were able to be analyzed and classified by using the developed system (ii) ANN were used in this system as a color space transformation model where the transformed value was later used for the classification purposes	De Oliveira et al. [108]
Dry beans	To classify different types of seed from the production	ANN, KNN, DT, SVM	(i) The classification of the beans was able to be done by all the ML algorithms with SVM which achieved the highest overall classification rates of 93.13% followed by the DT, ANN, and KNN where the classification rates were 92.52%, 91.73%, and 87.92%, respectively	Koklu & Ozkan [239]
Eggs	To predict the volume of eggs	ANN	(i) The volume of the eggs was predicted by a developed system with a good linear coefficient of 0.9738 with the actual volume and relative absolute error of 2.2078% which indicates that the developed system is an efficient model	Siswantoro et al. [240]
Figs	To classify fig fruits based on its visual features	Decision tree-FL	(i) Comparison was carried out between three different types of decision tree for the data obtained from CVS, and it was shown the REP decision tree had the highest value of *R* and lower RMSE values and hence was selected to be implemented in the fuzzy system (ii) The developed system was able to classify the fig fruits into five qualitative grades with a high accuracy	Khodaei & Behroozi-khazaei [241]
Fish	To identify the freshness of the fish	ANN, PCA	(i) The developed system was able to classify the freshness of fish with a success rate of 94.17% in the training set and 90.00% in the prediction set	Huang et al. [242]
Gluten-free cake	To develop a system for quality control of celiac-friendly products	FL	(i) The developed system was able to study the texture of the cake when different amounts of materials were added to it, and the optimal ingredients value suitable for the gluten-free cake were able to be determined	Rezagholi & Hesarinejad [243]
Lime	To predict the weight of Indian lime fruits	ANFIS	(i) Different clustering methods were fused with the ANFIS model to improve the accuracy in the classification system, and it was found that the fuzzy C-means clustering (FCM) was the best in predicting the weight of the sweet lime (ii) The developed system was able to predict the weight of the Indian sweet lime fruits accurately	Phate et al. [244]
Mango	To estimate the fruit mass of the mango	ANN	(i) The data obtained from the CVS was used as the input parameters in the developed ANN model, and the model was able to estimate the fruit mass successfully (ii) The developed system has the highest success rate of 97% and the efficiency coefficient of 0.99 by applying two input parameters or three input parameters	Utai et al. [245]
Mushrooms	To determine the appearance quality of mushrooms	ANN, FL	(i) The accuracy obtained by the image processing system was 95.6% (ii) The artificial neural network was able to determine the weight of the mushrooms, and the fuzzy logic used the data from the CVS and was able to determine the quality of the mushroom	Nadim et al. [246]
Passion fruits	To classify the passion fruits based on their ripeness level	Multi-class SVM (MCSVM)	The developed system was able to classify the ripeness level passion fruits with an accuracy of 93.3% within 0.94128 s	Sidehabi et al. [247]
Pork loin	To assess the quality of the pork loin according to the industry demand	SVM	(i) The model was able to predict the quality of the pork loin based on their color and quality attributes based on the industries’ demand	Sun et al. [248]
Potatoes	To develop a grading system for potatoes	FL	(i) Combination of CVS and fuzzy logic allows faster grading of the potatoes as well as reduces the cost required for the manual grading	Bhagat & Markande [249]
Rice	To carry out the qualitative grading of milled rice	FL	(i) The study was able to conclude that the developed hybrid system can be used in the processing industry for automatic grading of milled rice (ii) Comparison was done between the developed system and experts’ judgment, and around 89.80% overall confidence was obtained (iii) The fuzzy system has obtained 89.83% total sensitivity and 97.45% specificity for the quality grading of milled rice	Zareiforoush et al. [250]
Rice	To control the performance of rice whitening machines	FL	(i) The developed automatic control system had an average of 31.3% higher performance speed than that of a normal human operator, and there was an improvement in the quality of the output based on the decision made by the system (ii) The setup flexibility of the system allows alteration to be done according to the preference of each rice mill operator	Zareiforoush et al. [55]
Tea	To classify the Iranian green and black tea	DT-FL	(i) REP decision tree was shown to be more convenient compared to the J48 tree for developing a fuzzy classifier system (ii) The research successfully showed that DT-based fuzzy systems can be applied for automated intelligent classification of Iranian green tea and black tea	Bakhshipour et al. [251]
Tomato	To detect the maturity of the fresh tomato	ANN	(i) The maturity of the tomato was able to be detected by using the developed system with an accuracy of 99.31% and 1.2% of standard deviation	Wan et al. [252]
Vegetable seeds	To classify the vegetable seeds	FL	(i) The system can classify the two different types of seeds that look similar which are cauliflower seed and Chinese cabbage seed	Garcia et al. [253]

Near infrared spectroscopy (NIRS) is another technique in the food industry as there is no usage of chemicals and results can be obtained accurately as well as precisely within minutes or even continuously [114]. In addition, it is known to be non-destructive, cost effective, quick, and straightforward which makes it a good alternative for the traditional techniques which are expensive and labor intensive and consumes a lot of time [115]. The chemical-free method by NIRS makes it suitable to be used as a sustainable alternative since it will not endanger the environment or the human health. It has a wide range of quantitative and qualitative analysis of gases, materials, slurries, powders, and solid materials. Furthermore, samples are not required to be grounded when light passes through it and certain features or characteristics that are unique to the class of the sample are revealed by the spectra of the light. Complex physical and chemical information on the vibrational of molecular bonds such as C–H, N–H, and O–H groups and N–O, C–N, C–O, and C–C groups in organic materials can be provided by the spectra which can be recorded in reflection, interactance, or in transmission modes [114].

**Fig. 10:**
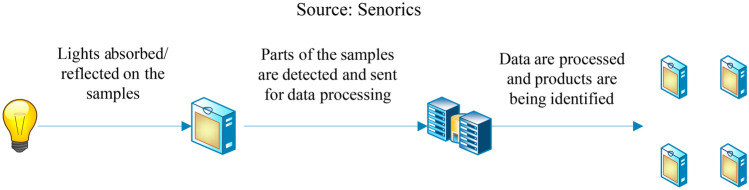
Basic working principle of CVS

The basic working principle for NIRS is shown in Fig. 10. Recently, NIRS has become an interest in food industries to inspect food quality, controlling the objective of the study and evaluating the safety of the food [114, 116–119]. Several researchers have applied the NIRS in food to obtain its properties for multiple reasons including determining the fatty acid profile of the milk as well as fat groups in goat milk [120]. Apart from that, it is able to aid in the prediction of salted meat composition at different temperatures [121] and in the prediction of sodium contents in processed meat products [122]. The detection and grading of the wooden breast syndrome in chicken fillet in the process line was also able to be performed by using the NIRS technique [123]. Not only that, it is proven to be efficient in determining the maturity of the avocado based on their oil content [124], predicting the acrylamide content in French-fried potato and in the potato flour model system [125], and determining the composition of fatty acid in lamb [126]. There has been a review conducted on the application of the ANN combined with the near-infrared spectroscopy for the detection and authenticity of the food [127]. The ability of the NIRS system in detecting the physical and chemical properties coupled with soft computing techniques such as ANN, FL, and ML allows the classification and prediction of the samples to be performed rapidly and accurately. Table 9 shows the application of NIRS coupled with AI techniques in the food industry.

**Table 9:** Application of NIRS with AI in food industries

Application	Objectives	AI technique	Important outcomes	References
Cheese	To determine the characteristics of controlled-processing cheese	PCA-ANN	(i) Sensory attributes of cheese have been determined with sufficient evidence on well-defined population which was known to be cost effective and useful method for quality control	Curto et al. [254]
Chicken meat	To classify chicken meat with respect to their quality grades	Decision tree (DTree)	(i) The assessment of the poultry meat was able to be done by using the spectral analysis and the classification using the DTree models (REPTree) had a better performance compared to the support vector machine and decision table model that were carried out for comparison purposes	Barbon et al. [255]
Chocolate	To develop a model for the quality assessment of chocolate	ANN	(i) NIRS was used to obtain the fingerprint of the chocolate using the five taste senses and fused with ANN for the prediction of chocolate quality based on chemical, physical, and sensory properties and accurate prediction was achieved by the developed model	Gunaratne et al. [256]
Civet coffee	To detect any alteration in the civet coffee	ANN, SVM, KNN	(i) The combination of trained ANN and NIRS method was able to discriminate the civet coffee from non-civet coffee successfully with an accuracy in the range of 95–100% (ii) The performance of classification using ANN was compared with other learning algorithms such as decision trees, discriminant analysis, SVM, KNN, and ensemble classifiers, and it was proven that ANN has better accuracy compared to the others	Arboleda [257]
Gelatin	To detect the adulteration in edible gelatin	ANN, SVM	(i) NIRS fused with both methods which were done separately was able to detect the adulteration in edible gelatin (ii) SVM model showed as the best recognition methods among the other models that were used with an accuracy of 100% meanwhile the recognition rate obtained by the ANN model was 97.44%	Zhang et al. [258]
Eggs	To determine the freshness of the egg	RBFNN, PCA	(i) The system was able to classify the egg freshness, and the model is appropriate to be implemented for the rough screening of the eggs	Aboonajmi et al. [259]
Food powders	To classify different types of food powders	SVM	(i) The system was able to classify the food powders varying of whole wheat flour, organic wheat flour, tapioca starch, corn starch, and rice flour with a very high accuracy rate of 100%	Mohamed et al. [260]
Keemun black tea	To develop a model for the discrimination of different grades of keemun black tea in China	ANN, least square SVM (LSSVM), Random Forest	(i) Three different methods which are ANN and LSSVM were used to develop the model and all the methods were able to distinguish the different grades of keemun tea (ii) It was identified that the LSSVM method has a higher performance and predictivity compared to the rest	Ren et al. [261]
Meat	To create a system for the detection of meat spoilage	AFLS	(i) The AFLS model was able to classify the meat into three classes which are fresh, semi-fresh, and spoiled by using the data provided by the FTIR spectrometer (ii) The model achieved a high percentage with a value of 95.94% of correct classification overall which indicates that it can be an effective tool for the detection of meat spoilage	Alshejari & Kodogiannis [262]
Olive oil	To detect the adulteration in olive oils	SVM	(i) The fusion of NIRS and Raman spectral data could identify the adulterated olive oils effectively and the SVM model was able to predict the dopant contents in olive oil accurately	Xu et al. [263]
Pears	To determine the soluble solid contents in pear	ELM	(i) The proposed successive projection algorithm and extreme learning machine (SPA-ELM) was able to predict the contents better than the conventional PCA-ELM method	Lu et al. [264]
Rice	To classify the rice according to the compositions and processing parameters	RF, PCA, LDA, PLS	(i) All the ML techniques were able to identify and classify the rice based on its composition (amylose-based, glycemic index) and the hydrothermal treatment severity with a good performance	Rizwana & Hazarika [265]
Wheat flour	To predict the wheat flour quality using the NIRS	Multitarget coupled with SVM, RF	(i) The use of multitarget over partial least squares coupled with machine learning algorithm offers more advantage for the parameter prediction from NIRS (ii) The prediction using random forest overcomes the performance of SVM	Barbon Junior et al. [266]
White asparagus	To predict the origin of the asparagus and distinguish the German from imported products	SVM	(i) The linear SVM could predict the country of origin of white asparagus with an accuracy of 89% and also was able to distinguish the German and non-German products	Richter et al. [267]

## Summary on the Application of AI in the Food Industry

From the review so far, it can be shown that AI has been used for various reasons in food industries such as for detection, safety, prediction, control tool, quality analysis, and classification purposes. Ranking of sensory attributes in the foods can be done easily by using the FL model. Not only that, fuzzy logic can be used for classification, control, and non-linear food modeling in the food industry. ES is widely used in the food industry for decision-making process. On the other hand, ANN model is applied widely in the food industry for prediction, classification, and control task as well as for food processing and technology. The supervised ANN method has the ability to learn from examples which allows for the prediction process to be done accurately. Meanwhile, the unsupervised method of ANN is found to be more common for the classification task. Another method that has been utilized for the prediction and classification of the food samples is by using the machine learning (ML) method. ML can be used in solving complicated tasks which involves a huge amount of data and variables but does not have pre-existing equations or formula. This method is known to be useful when the rules are too complex and constantly changing or when the data keep changing and require adaptation. Furthermore, the adaptive neuro fuzzy inference system (ANFIS) is another hybrid AI method that can be used to solve sophisticated and practical problems in the food industry. However, decent data are required for the model to learn in order to perform well. In addition to that, this model is useful for solving analytical mathematical models in the food industry such as studies involving mass and heat transfer coefficients. ANFIS is recommended to be used when complex systems where time-varying processes or complex functional relationships and multivariable are involved. Apart from that, it can be used in descriptive sensory evaluation.

These AI algorithms can be combined with other sensors such as the electronic nose, electronic tongue, computer vision system, and near infrared spectroscopy to glean the data from the samples. Both the E-nose and E-tongue have shown to enhance the quality characteristics in comparison to the traditional detection approach [128]. E-nose can be used to sense the odors or gases while the E-tongue can be applied for the identification of the organic and inorganic compounds. Studies involving the examination and drawing out the features of the samples like shape, color, defects, and size can be carried out by using the CVS sensors. NIRS can be utilized to determine the properties or contents in the samples. The data obtained from these sensors is then merged with the AI algorithms and utilizing their computing strengths to accomplish the desired studies.

## Advantages and Disadvantages of AI

AI has been used widely in the industry as it offers a lot of advantages compared to the traditional method. All the algorithms are known to be accurate and reliable, but careful selection should be made by considering the advantages and limitations of the algorithms. The different algorithms have their own strengths and weakness, hence choosing them for a particular application in the food industry needs to be looked on a case-to-case basis. The guideline to choose the most appropriate method is given in the next section. The benefits and constraints that each of the algorithm exhibits are explained briefly in Table 10.

**Table 10:** Advantages and limitations of AI algorithms

AI algorithm	Advantages	Limitations
ES	(i) Reliable and understandable (ii) Highly responsive (iii) High performance (iv) Error rate is lower than human errors (v) Better use of production capacities	(i) The construction and designing of the ES are expensive and rare as it requires expert engineers (ii) Vocabulary utilized by the experts is limited and often is difficult to be understood
FL	(i) Imprecise, incomplete, and uncertain information can be solved (ii) Simpler and direct results can be obtained (iii) Accountable, noise tolerant, and robust to disturbances (iv) Faster interpretation than ANN and support vector machine method (v) The knowledge base can be extended easily with the extension of the rules (vi) Saves costs and time (vii) Can improve the quality and safety of the products	(i) Generalization is not possible as it can only deduce the given rules (ii) Sometimes requires the knowledge of an expert in creating the rules
ANN	(i) Able to model complex functions or problems accurately and easily (ii) Accurate, robust to disturbances, and noise tolerant (iii) Has the ability to learn from the patterns or examples (iv) Has the generalization ability (v) Affordable, noise tolerant, easy, and flexible method (vi) Solving non-linear problems are more appropriate by using this method (vii) Useful as prediction, classification, and control tool	(i) The performance of the model is hard to be explained compared to the others as it appears as a black box model (ii) Requires more time compared to the FL as the suitable number of layers should be determined (iii) Need sufficient and reliable data
ANFIS	(i) Able to merge details from various resources (ii) Noise tolerant, accurate, and effective method in solving complex problems (iii) It has a higher performance compared to ANN and FL methods (iv) Possesses the benefits from both ANN and FL method (v) Classification and prediction tasks can be done more conveniently (vi) Able to save time and cost overall in general compared to manual methods	(i) The data available should be reliable to avoid any confusion or misinformation during the training process as it will affect the final results

## Guidelines on Choosing the Appropriate AI Method

**Fig. 11:**
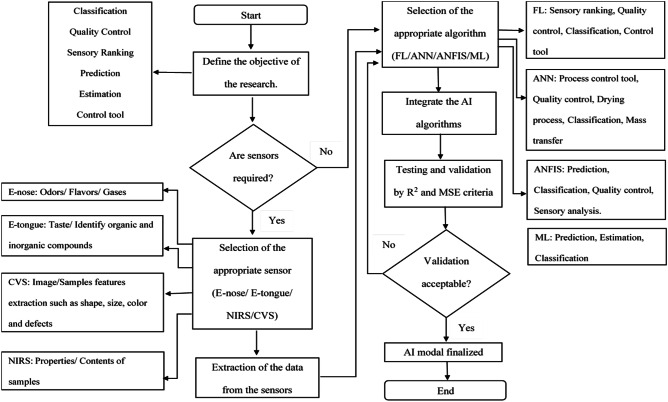
Flowchart for developing AI model

Selecting the appropriate algorithm is important when developing the AI model as it can aid the user to attain an accurate, rapid, and cost-saving results. Therefore, a guideline given in Fig. 3 is an important asset prior to achieving best performances in a case study. The primary step in the selection process is that users should define and finalize the objective of using AI in their research or implementation. Prediction, classification, quality control, detection of adulterants, and estimation are among the common objectives of AI applications in the food industries. Next, decision should be made whether sensors such as E-tongue, E-nose, CVS, and NIRS are required to collect the sampling data or not for collecting the data from the samples. Normally, integration with those sensors is conducted to obtain the parameters and characteristics of the samples to be included in the AI algorithms for sample testing purposes. Upon deciding the necessity of the sensors, users should compare and choose the fitting algorithm with respect to their study. Among the most common AI algorithms that have been employed include the FL, ANN, ANFIS, and ML methods. ANFIS has shown to have a higher accuracy, but the complexity of the model makes it less favorable compared to the other algorithms. It is advisable for the users to determine the complexity of the research in selecting the most appropriate algorithm for their studies. Once the selection of the algorithm has been confirmed, the data available are integrated with the AI algorithms. Finally, the testing and validation based on *R*^2^ and MSE are done to analyze the performance of the established model. The AI model has been created successfully once the validation is accepted; otherwise, users should return to the previous step and reselect the algorithm. Figure 11 shows the guideline in choosing and development of the AI model in food industry application.

## Trends on the Application of AI in the Food Industry in the Future

**Fig. 12:**
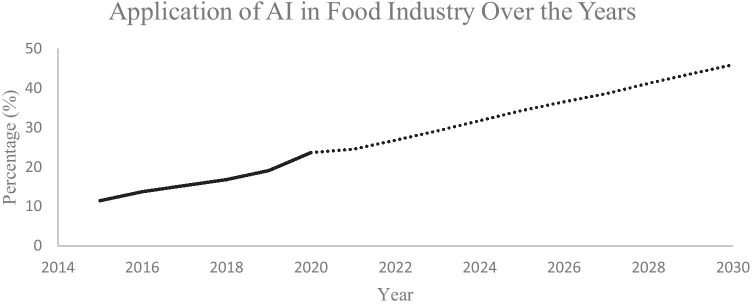
Application of AI in the food industry

The overall trend on the application of AI in the food industry is shown in Fig. 12. From the studies within the past few years, the usage of the AI methods has been observed to increase from 2015 to 2020 and is predicted to rise for the next 10 years based on the current trends. Among the rising factors for the application of AI in the food industry is the introduction of Industrial Revolution 4.0 (IR 4.0). The merging of technologies or intelligent systems into conventional industry is what is known as IR 4.0 and can also be called smart factory [129, 130]. AI which is categorized under the IR 4.0 technologies focuses on the development of intelligent machines that functions like the humans [131]. IR 4.0 makes a great impact in the product recalls due to the inspections or complains in the food industries. The implementation of the AI integrated in the sensors able to detect the errors during the manufacturing process and rectify the problems efficiently. Apart from that, IR 4.0 also plays a big role in the human behavior as consumers in the twenty-first century often discover information regarding the foods in the internet. The rising concerns on the food quality allow more usage of AI as they are able to enhance the quality of the food and aids during the production process. The highest amount of application of AI in the food industry was seen in the year 2020 as more researchers are carrying out studies using the AI method, and it is believed to continue rising for the upcoming years due to increasing in food demand and the concern on the safety of the foods which are being produced.

**Fig. 13:**
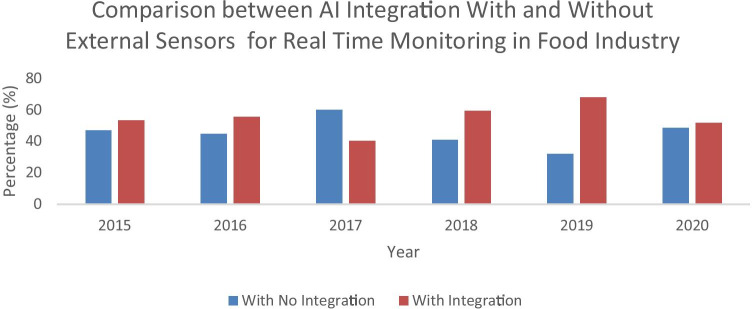
Comparison between integration of AI for real-time monitoring in the food industry

The comparison between the AI integration with and without sensors for real-time monitoring in the food industry is displayed in Fig. 13. Integration with external sensors has a higher percentage compared to those without the integration of the sensors in the food industries. The purpose of external sensors was to obtain the data from the samples which are then employed into the AI algorithms to carry out various tasks such as classification, prediction, quality control, and others that have been stated earlier. However, the data collection for the year 2017 showed that the percentage for the AI without the external sensors is greater than that with integration with the sensors. This is due to the high amount of research which was conducted without using the external sensors which are listed in this paper. Based on the evaluation carried out during this study, it was found that a high amount of research was done on the integration of CVS sensors with the AI methods. It is explainable as CVS sensors are able to provide important parameters such as the shape, size, colors, and defects which are essential for the quality control in the food industry. However, the integration of the system is mainly dependent on the objectives of the researcher and the industrial players and the availability of the data.

In short, as the AI world is heading towards 2.0 [132], it can be predicted that the rise in the usage of AI in the food industry is definite and inevitable because of the advantages that they can offer such as saving in terms of time, money, and energy as well as the accuracy in predicting the main factors which are affecting the food industries. Apart from that, in the recent pandemic situation due to the Covid-19 virus, it is predicted that more companies will opt for the usage of AI in their industries to cut down the costs and boost the performance of their company. There have been reports by some of the SMEs that their earnings have dropped and some SMEs have claimed that they could only survive for about 1 to 3 months. The high demand of food and the tight standard operating procedure in the companies during the pandemic situation will encourage the industry players to find an alternative to their problems and AI will be one of them to ensure a smooth operation.

## Conclusion and Future Outlook

In conclusion, AI has been playing a major role in the food industry for various intents such as for modeling, prediction, control tool, food drying, sensory evaluation, quality control, and solving complex problems in the food processing. Apart from that, AI is able to enhance the business strategies due to its ability in conducting the sales prediction and allowing the yield increment. AI is recognized widely due to its simplicity, accuracy, and cost-saving method in the food industry. The applications of AI, its advantages, and limitations as well as the integration of the algorithms with different sensors such as E-nose and E-tongue in the food industry are critically summarized. Moreover, a guideline has been proposed as a step-by-step procedure in developing the appropriate algorithm prior to using the AI model in the food industry–related field, all of which will aid and encourage researchers and industrial players to venture into the current technology that has been proven to provide better outcome.
